# Double-Blind Placebo-Controlled Randomized Study of Sporopollenin Exine (SpEC) Fragrance Encapsulation

**DOI:** 10.3390/pharmaceutics18050609

**Published:** 2026-05-17

**Authors:** Mariam Murad, Pearl Wasif, Laura Dempsey, G. Roshan Deen, Alexandra E. Butler, Stephen L. Atkin

**Affiliations:** 1 School of Postgraduate Studies and Research, Royal College of Surgeons in Ireland, Medical University of Bahrain, Busaiteen 228, Bahrain; mmurad@rcsi.com (M.M.); pwasif@rcsi.com (P.W.); satkin@rcsi.com (S.L.A.); 2 Data Science Centre, School of Population Health, Royal College of Surgeons of Ireland, D02 YN77 Dublin, Ireland; lauradempsey@rcsi.ie; 3 School of Medicine, Royal College of Surgeons in Ireland, Medical University of Bahrain, Busaiteen 228, Bahrain; rdeen@rcsi.com

**Keywords:** *L. clavatum*, SpECs, fragrance, pollen shells, sporopollenin, clinical trial

## Abstract

**Objective**: Sporopollenin exine capsules (SpECs) have been used to encapsulate active pharmaceutical agents for oral drug delivery. This study investigated whether fragrance encapsulated within SpECs prolonged perceived fragrance intensity compared with fragrance oil alone. **Methods**: A double-blind, placebo-controlled, randomized pilot study was conducted (clinical trial number: NCT07383337); ten healthy female participants (mean age 35.4 ± 5.6 years) received fragrance with SpEC-encapsulated fragrance (SpECs) on one wrist and fragrance alone (control) on the contralateral wrist. The fragrance intensity was assessed using a visual analogue scale (0–10) by both the participants and an independent blinded reviewer at baseline and after 2, 4 and 8 h. Paired Wilcoxon signed-rank tests and linear mixed-effects models were used for analysis. In vitro cytotoxicity was assessed using an ATP viability assay in human bronchial epithelial (BEAS-2B) cells exposed to SpECs or raw *Lycopodium clavatum* spores. **Results**: There were no significant differences between the formulations at baseline. From 2 h onward, SpECs was associated with significantly a higher fragrance intensity compared with the control for both participant-rated (*p* = 0.03 at 2 and 4 h; *p* = 0.005 at 8 h) and reviewer-rated assessments (*p* = 0.02 at 2 h; *p* = 0.01 at 4 h; and *p* = 0.008 at 8 h). Mixed-model analyses suggested a greater decline in intensity for control at 8 h for reviewer-rated assessments. In vitro, raw spores significantly reduced cell viability (as an indicator of potential allergenicity), whereas SpECs did not differ from control. **Conclusions**: Fragrance encapsulation within SpECs significantly prolongs measured fragrance intensity with no evidence of cytotoxicity. These findings support the potential of SpECs as a safe and effective sustained-release platform for topical fragrance formulations.

## 1. Introduction

Plant-derived biomaterials have attracted increasing attention in recent years due to their unique structural, physicochemical, and functional properties, which offer innovative solutions across pharmaceutical, biomedical, and cosmetic applications [1,2,3]. Among these, sporopollenin—the principal biopolymer forming the outer exine layer of pollen grains and spores—has emerged as a particularly promising material owing to its exceptional chemical stability, mechanical resilience, and resistance to enzymatic degradation [4,5]. Sporopollenin is composed of a highly cross-linked macrocyclic polymer network with polyhydroxylated tetraketide-like monomers and conjugated phenolic structures, which contribute to its robustness and redox activity [5,6]. These features make lipophilic sporopollenin one of the most chemically inert and durable natural biomaterials known.

Sporopollenin exine capsules (SpECs) are obtained by removing the internal cytoplasmic content of pollen or spores, resulting in hollow, porous microcapsules that retain the highly structured lipophilic exine shell that provides a hydrophobic internal environment [7,8]. These capsules typically contain nano-scale channels (~40 nm) that allow for the loading and controlled release of a wide range of active compounds [4,8]. The size, morphology, and surface topology of SpECs are species-specific, with *Lycopodium clavatum*-derived capsules exhibiting a highly uniform spherical structure with characteristic serrated coronate features [2]. This structural uniformity and surface complexity provide advantages in terms of reproducibility, loading capacity, and potential bioadhesive properties [9,10,11].

A key advantage of SpECs lies in their ability to encapsulate lipophilic compounds within their hydrophobic internal environment. Previous studies have demonstrated the successful loading and sustained release of bioactive molecules such as omega-3 fatty acids and vitamin D, with improved bioavailability attributed to mucoadhesion and controlled release mechanisms [10]. The release of encapsulated compounds is thought to occur through diffusion and mechanical shear, whereby external forces applied, such as pressure from rubbing, to the capsule facilitate the gradual expulsion of the contents through the nanochannels [7,8]. This controlled release behaviour positions SpECs as an attractive delivery platform for applications requiring prolonged exposure or sustained activity.

In addition to their functional advantages, SpECs offer important safety benefits. While intact pollen and spores are well recognized for their allergenic potential due to the presence of proteins and other bioactive molecules, the extraction process used to generate SpECs removes all internal biological material, yielding protein-free shells composed solely of sporopollenin [12]. This significantly reduces the risk of immunogenicity and has been supported by recent studies demonstrating minimal IgE reactivity and the good biocompatibility of sporopollenin-derived materials [12,13]. Furthermore, SpECs have shown favourable toxicity profiles in vitro and in vivo, reinforcing their suitability for biomedical and topical applications.

Beyond pharmaceutical delivery, there is growing interest in the application of SpECs within the cosmetics and personal care industries. Fragrance formulations, in particular, present a significant challenge due to the volatile nature of fragrance compounds, which often leads to rapid evaporation and a decline in their perceived intensity over time. Conventional approaches to prolong fragrance longevity include the use of fixatives or encapsulation within synthetic polymers; however, these strategies may have limitations related to stability, skin compatibility, or environmental impact. In this context, SpECs represent a potentially novel, natural, and biodegradable alternative for fragrance encapsulation and sustained release [1,2,3].

The unique structural characteristics of SpECs suggest that they may function as micro-reservoirs for fragrance oils, protecting volatile components from immediate evaporation while enabling gradual release over time. Their mechanical robustness and elasticity allow them to withstand external forces without rupture, while repeated shear interactions—such as those occurring during normal skin movement—may facilitate the progressive release of encapsulated fragrance [7]. Additionally, the potential bioadhesive properties of the exine surface may enhance retention on the skin, further contributing to prolonged fragrance perception [10,11].

Despite these promising attributes, there is currently a lack of human studies evaluating the effectiveness of SpECs in fragrance delivery. Most existing research has focused on pharmaceutical or nutraceutical applications, with limited exploration of their role in cosmetic formulations. Furthermore, while the safety profile of SpECs has been demonstrated in preclinical studies, comparative assessments against raw pollen or spores in the context of topical exposure remain limited.

Taken together, the unique structural resilience, tuneable porosity, and inherent biocompatibility of SpECs position them as a transformative platform for next-generation topical delivery systems. We hypothesize that SpECs function as mechanically responsive micro-reservoirs that modulate the spatiotemporal release of volatile fragrance compounds through a combination of diffusion-limited transport and shear-triggered expulsion, thereby extending olfactory perception beyond the constraints of conventional formulations. Furthermore, we propose that the species-specific surface topology and potential bioadhesive properties of *Lycopodium clavatum*-derived SpECs enhance retention at the skin interface, reducing volatilisation losses and enabling sustained fragrance intensity. This dual mechanism—controlled release coupled with enhanced surface residence—represents a paradigm shift from passive fragrance evaporation to an active, material-driven delivery system. In this context, the present study was designed as a first-in-human, proof-of-concept investigation to evaluate whether SpEC-based encapsulation can significantly prolong fragrance perception compared with standard formulations, while maintaining a favourable safety profile.

## 2. Materials and Methods

### 2.1. Materials

The fragrance oil Arabian Rose was purchased from Arabian Oud, Riyadh, Saudi Arabia. *Lycopodium clavatum* spores were obtained from Sporomex Ltd. (Driffield, UK) from which the SpECs were prepared. Sodium hydroxide, acetone (analytical grade), and hydrogen peroxide were obtained from Sigma-Aldrich (Gillingham, UK) and used as received.

### 2.2. Preparation of SpECs

SpECs were prepared according to the two-step extraction process (patent WO2010004334A3 Whitened Exine Shells). *Lycopodium clavatum* spores (200 mg) were treated sequentially with acetone and sodium hydroxide to remove the cytoplasmic materials with whitening accomplished using hydrogen peroxide. A final step involved the acidification of the outer surface with 10% acetic acid (e.g., white vinegar) to optimize the whiteness of the exines, followed by washing in hot water and drying. Combustion elemental analysis found carbon: 67.9%; hydrogen: 79.19%; and nitrogen: 0.0%.

### 2.3. Loading of Fragrance Oil into SpECs

**Figure 1:**
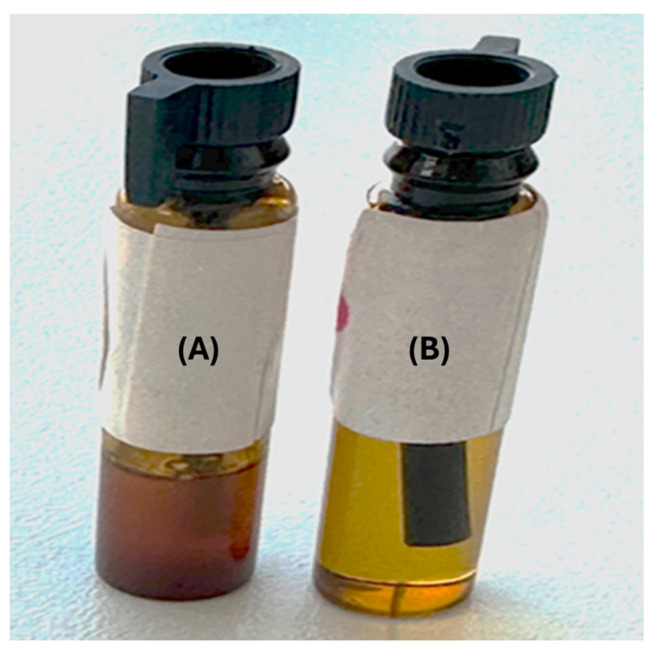
Unblinded fragrance preparation (**A**) (fragrance encapsulated in Sporopollenin exine capsules (SpECs)), showing the turbidity of the fragrance oil with the addition of the SpECs that could not be seen with an opaquer bottle, and fragrance preparation (**B**) (fragrance alone).

**Figure 2:**
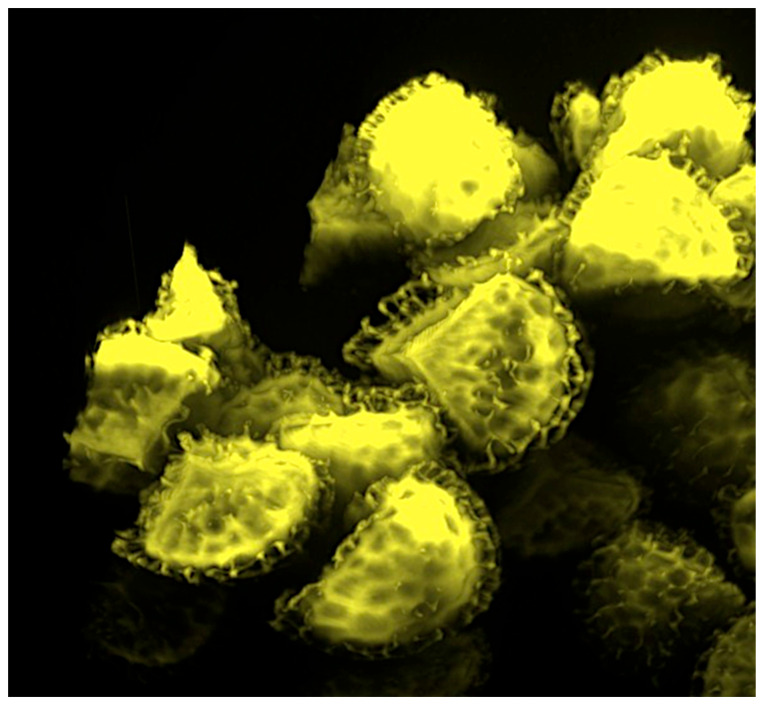
Confocal microscopy images (×60) of fragrance encapsulated within Sporopollenin exine capsules (SpECs).

The fragrance oil (200 mg) was added to SpECs (200 mg), mixed and put under vacuum (enhancing the rapidity of filling) for 1 h. A preparation of 40 mg/mL of fragrance-loaded exines in the fragrance oil was finally prepared. The appearance of the unblinded preparations is shown in Figure 1 and the fragrance (100%)-filled SpECs are shown by confocal microscopy in Figure 2.

### 2.4. Randomized Trial Studies

Ten female subjects were recruited by means of adverts (mean age, 35.4  ±  5.6 years). The exclusion criteria included upper respiratory tract infections, any cause of anosmia, a history of allergy to fragrances or any skin allergies.

Two preparations, one with 40 mg/mL fragrance-loaded SpECs within the fragrance oil (SpECs) and the other with fragrance oil alone (control), were contained in identical opaque bottles that had been randomized by a third person who took no further part in the study. A computer randomisation table was used to determine which preparation was applied to the right wrist and the other to the left wrist for each subject using a plastic perfume applicator for each bottle, which was dipped once into the bottle and applied by the research assistant. The subjects were asked to use a visual analogue scale (zero to ten with ten being the most fragrant) for each wrist at baseline and after 2, 4 and 8 h.

An independent evaluator of the fragrance strength (who was consistent across all assessments and who was not involved in the sample preparation or application), evaluated each wrist at baseline, 2, 4 and 8 h using the visual analogue scale (zero to ten). All participants provided written informed consent. The study was performed in accordance with the declaration of Helsinki and was approved by RCSI Bahrain ethics committee (number REC/2025/271/20-May-2025: ethics approval date 20 May 2025). The trial registration number is NCT07383337.

### 2.5. Scanning Electron Microscopy (SEM)

The SpEC sample was imaged on a scanning electron microscope (Invenso SEMoscope, Cambridge, MA, USA) with an accelerating voltage of 6–20 kV. The sample was sputtered with gold (10 nm layer) for 2 min on a Inovenso SPT-20 sputter (Cambridge, MA, USA) operating at a voltage of 15 kV. The electron micrograph is shown in Supplemental Figure S1. The external architecture of the SpEC was intact and uniform.

### 2.6. Fourier Transform Infra-Red (FTIR) Spectroscopy

FTIR spectra of the raw *Lycopodium clavatum* and SpEC were recorded in the wavenumber ranger of 4000–500 cm^−1^ with a spectral resolution of 0.5 cm^−1^, using a Bruker Alpha-FT-IR spectrophotometer (Ettlingen, Germany). The samples were mixed with dry potassium bromide (Sigma-Aldrich, spectroscopic grade) and made into a thin transparent disc. The spectra are shown in Supplemental Figures S2 and S3. Significant changes in the absorption bands of SpEC indicated chemical modifications compared to the raw spores.

### 2.7. Confocal Microscopy

Confocal fluorescence imaging was performed using a four-laser scanning confocal microscope. Images confirmed the internal morphology was intact and the SpECs were empty.

## 3. In Vitro Studies for Allergenicity

### 3.1. Cell Culture

Human epithelial BEAS-2B cells (ATCC CRL-3588, Manassas, VA, USA), a cell line used typically in toxicological studies, were revived as per ATCC recommendation. The cells were maintained by serial passage in DMEM (Sigma D5796, Darmstadt, Germany) with 10% fetal calf serum (Gibco 10270-106, New York, NY, USA) and 1% Penicillin–streptomycin (Sigma P4333, Darmstadt, Germany) in Nunclon Delta T75 flasks (ThermoFisher 156,499, Waltham, MA, USA). The cells were subcultured every third day before they reached confluent growth and terminal differentiation.

### 3.2. SpEC and L. clavatum Spore Suspensions

The SpEC preparation and raw intact *L. clavatum spores* available in dry powder form were weighed and reconstituted in sterile PBS (Gibco 10,010,023, New York, NY, USA) to a concentration of 10 mg/mL. The preparations were kept on a shaker and rocked gently overnight to ensure the soakage and even dispersion of the SpEC preparations and spores. Overnight-soaked preparations settled to the bottom of the solution. The preparations were then sterilized by autoclaving in 121 °C at 15 psi for 15 min and stored in room temperature until further use.

### 3.3. Adenosine Triphosphate (ATP) Assay for Cell Viability

In total, 2 × 10^4^ BEAS-2B cells were seeded per well in a 96-well flat-bottom white plate (Corning 3917, New York, NY, USA) and incubated at 37 °C overnight to obtain a confluent monolayer mimicking the epithelial surface. Each of the SpEC/*L. clavatum* spore preparations were dispersed by pulse vortexing and were added to the corresponding wells to achieve a concentration of 1 mg/mL in the well. Silica gel granules (Sigma 717,185 Darmstadt, Germany) at the same concentration and an untreated cell control were used as the negative controls. The plate was incubated at 37 °C overnight to allow the test substances to settle on the cell monolayer. The next day, the supernatant culture medium was removed by aspiration and treated with the ATP Determination Kit (ThermoFisher A22066, Waltham, MA, USA) as per the manufacturer’s instructions. The luminescence was read in CLARIOstar Plus (BMG Labtech, Ortenberg, Germany) with the inbuilt QUICK LUM protocol. A standard curve was plotted, and the luminescence signals were translated to ATP concentrations in picomoles. Each sample was tested in triplicate and the mean value was taken as the final ATP concentration.

### 3.4. Statistics

No published studies are available that could facilitate performing a power calculation; therefore, we undertook a hypothesis-generating exploratory pilot study with ten participants in a within-subject design to obtain preliminary estimates of effect size and variability, and to inform future adequately powered trials. To explore initial between group differences, differences between Formula A (SpECs) and Formula B (control) at each timepoint were assessed using Wilcoxon signed-rank tests, an appropriate non-parametric method for paired data. Separate analyses were conducted for participant-rated and reviewer-rated intensity scores at each timepoint (0, 2, 4 and 8 h). To expand on the Wilcoxon signed-rank tests, and to explore any potential interactions between formula and time, a linear mixed model for repeated measures was run for both the participant data and reviewer data. The intensity score was used as the dependent variable, with formula, timepoint, and formula x timepoint interaction used as fixed effects and subject as random effect. This method analyses changes over time but also accounts for individual differences allowing us to see both overall trends and group-specific effects over multiple timepoints. Statistical analysis was performed using SAS version 9.4 and R Studio V 4.4.2.

## 4. Results

The data from the pilot study are summarized in Table 1 (participant-rated intensity scores) and Table 2 (reviewer-rated intensity scores). Groups A (SpECs: fragrance-loaded SpECs) and B (control: fragrance alone) represent the two fragrance formulations tested; however, both groups comprise the same ten participants.

**Figure 3:**
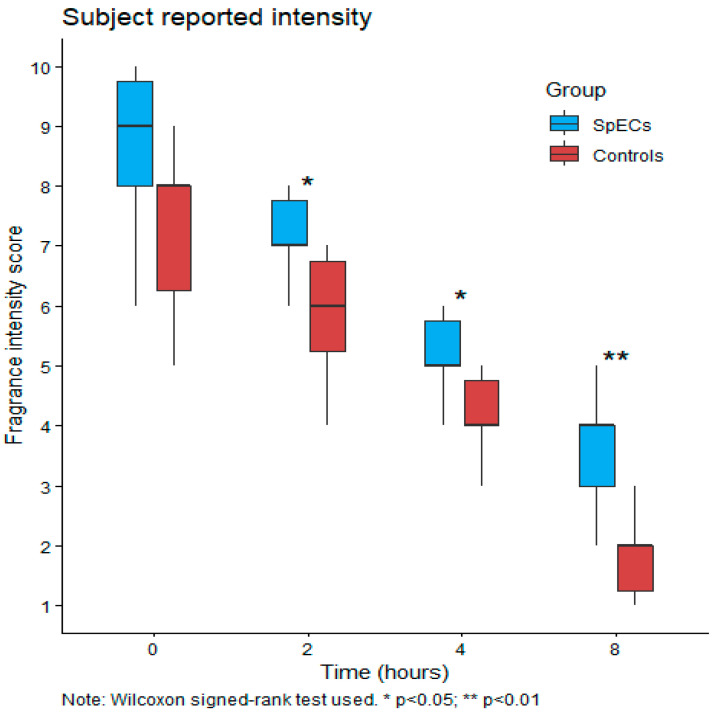
Boxplots of subject-reported intensity over time.

**Figure 4:**
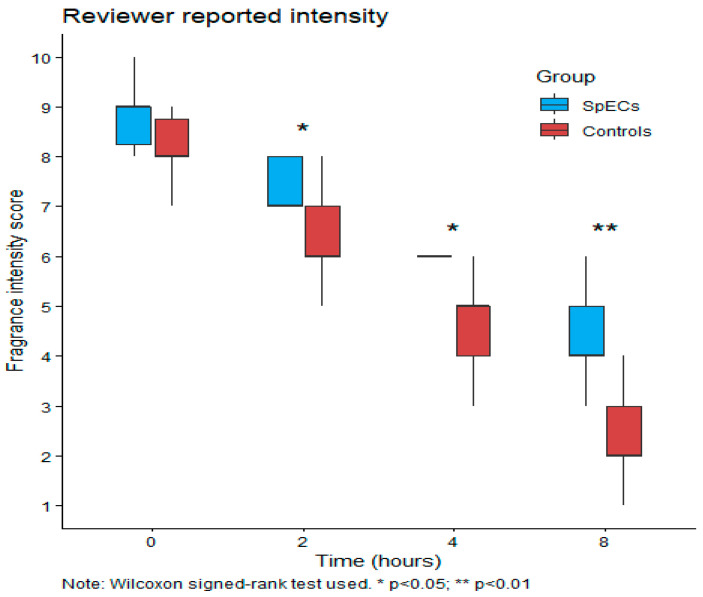
Boxplots of reviewer-reported intensity over time.

Differences between SpECs and control at each timepoint are summarized in Figure 3 (participant ratings) and Figure 4 (reviewer ratings). For participant-rated intensity, there was no statistically significant difference between formulations at baseline (*p* = 0.08). However, at 2 h (*p* = 0.03), 4 h (*p* = 0.03), and 8 h (*p* = 0.005), SpECs had significantly higher intensity scores than control.

For reviewer-rated intensity, no significant difference was observed at baseline (*p* = 0.3). From 2 h onwards, SpECs were consistently rated as more intense than control, with statistically significant differences at 2 h (*p* = 0.02), 4 h (*p* = 0.01), and 8 h (*p* = 0.008).

The linear mixed model for subject-reported scores showed evidence of a difference at baseline between the groups (*p* = 0.02). At baseline, participants rated control as being less intense than SpECs (on average 1 point less).

As expected, intensity over time decreases in both groups, and the rate of decline in intensity over time was similar for both formulations. There was no significant group * time interaction, suggesting there is no evidence that the way the two groups changed over time differed.

The linear mixed model for reviewer-reported scores showed no evidence of a difference at baseline between the groups (*p* = 0.17). As expected, intensity over time decreases in both groups overall. There was a significant group * timepoint interaction at 8 h (*p* = 0.01). This shows that control significantly decreases from baseline at 8 h when compared with SpECs (on average −1.3 points less).

**Figure 5:**
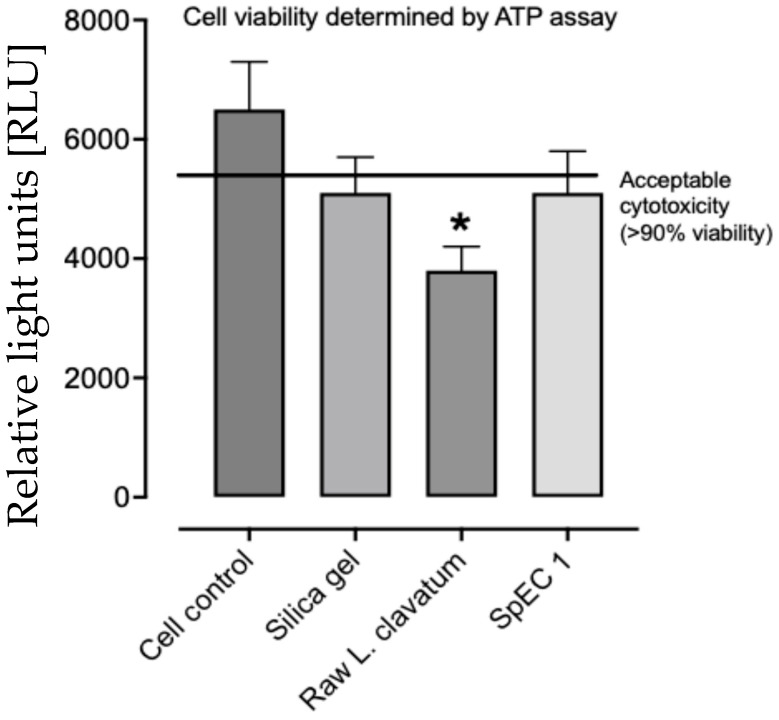
In vitro cytotoxic assay for allergenicity. The raw *Lycopodium clavatum* shows a decrease in cell viability compared to control compared to the Sporopollenin exine capsules (SpECs) that did not differ from the control. * *p* < 0.01 versus cell control.

The in vitro studies on cell cytotoxicity (as an indicator of potential allergenicity) showed that, compared to the cell control, there was a significant decrease in cell viability for the raw *L. clavatum* spores, but that the SpECs did not differ to the control as shown in Figure 5.

## 5. Discussion

This double-blind, randomized pilot study demonstrates that fragrance encapsulated within sporopollenin exine capsules (SpECs) provides a sustained fragrance release compared with fragrance oil alone, with significantly greater perceived intensity, persisting up to 8 h after application. Importantly, these findings were consistent across both participant-reported outcomes and blinded independent reviewer assessments, strengthening the robustness of the observed effect. In parallel, in vitro cytotoxicity testing confirmed that SpECs are non-toxic to human epithelial cells, in contrast to raw *Lycopodium clavatum* spores, supporting their suitability for topical human use.

The prolonged fragrance intensity observed with SpEC encapsulation is consistent with the unique physicochemical properties of sporopollenin. SpECs are highly cross-linked, chemically inert microcapsules with a porous architecture penetrated by nano-scale channels that allow for the loading and controlled release of lipophilic compounds [1,7,8]. Fragrance oils, which are predominantly hydrophobic, are well suited to sequestration within the sporopollenin matrix, where they are protected from rapid volatilization and environmental degradation. Mechanical shear generated by skin movement is thought to gradually expel the encapsulated fragrance through these nanochannels, resulting in a slow, sustained release rather than the rapid dissipation typically observed with conventional fragrance formulations [1,8,14].

This mechanism aligns with prior work demonstrating controlled release of lipophilic bioactives such as omega-3 fatty acids and vitamin D from SpECs, where prolonged bioavailability was attributed to mucoadhesive properties and shear-triggered release [9,10]. The serrated coronate surface topology characteristic of *L. clavatum*-derived SpECs may further enhance adhesion to the skin surface, reducing loss through evaporation or transfer and contributing to the extended sensory perception observed in this study [7,11,13]. In vitro experiments to investigate the bioadhesive properties of SpECs have been undertaken to compare them with examples of other known mucoadhesive materials, Carbopol and chitosan, using detachment force and the work of adhesion of empty SpECs using a Stable Micro Systems Analyser [10]. Overall, either in powder or tablet form, the SpECs showed better adhesive properties than chitosan, and was similar to Carbopol [10]. The absence of a strong formula-by-time interaction in the participant-reported scores suggests that while both formulations decline over time, the SpEC-containing formulation consistently maintains a higher intensity across multiple post-application timepoints.

A key strength of the present study is the use of both subjective (participant) and objective (blinded reviewer) fragrance assessments. Fragrance perception is inherently subjective and influenced by individual olfactory sensitivity, adaptation, and expectation. The concordance between participant-rated and reviewer-rated outcomes provides reassurance that the observed differences are attributable to the formulation rather than perceptual bias. Furthermore, the within-subject crossover design, in which each participant served as her own control, minimized inter-individual variability and enhanced the sensitivity of the analysis despite the small sample size, an approach appropriate for hypothesis-generating pilot studies [15].

Safety is a critical consideration for any topical delivery platform, particularly one derived from pollen or spores, which are widely recognized allergens. Raw pollen and spores contain allergenic proteins localized within the cytoplasmic contents and on the shell surface, and exposure can elicit inflammatory and allergic responses [12,16]. The SpEC extraction process removes all internal biological material, yielding protein-free shells composed solely of sporopollenin [17]. The in vitro findings presented here reinforce this distinction, demonstrating that raw *L. clavatum* spores significantly reduced epithelial cell viability, whereas SpECs showed no cytotoxic effect and were indistinguishable from the untreated controls. These results are consistent with prior reports indicating that SpECs are non-allergenic and biocompatible, including recent immunoreactivity profiling studies showing minimal IgE interaction with sporopollenin-derived biomaterials [12,17]. In addition, oral animal studies on differing SpECs have been undertaken that showed no inflammatory response or tissue activity [18] and human oral studies have been undertaken with these SpECs for the delivery of vitamin D and fish oils [9,10], further emphasizing their safety.

From a translational perspective, the ability of SpECs to function as fragrance reservoirs offers several potential advantages for the fragrance and personal care industries. Prolonged fragrance perception could reduce the need for higher fragrance concentrations, frequent reapplication, or synthetic fixatives, thereby improving consumer experience and potentially reducing irritancy. Encapsulation within SpECs may also protect volatile fragrance components from oxidation, photodegradation, and environmental loss, enhancing formulation stability and shelf life [1,19,20]. Moreover, as SpECs are derived from natural plant sources and are biodegradable, they may represent a more sustainable alternative to synthetic microcapsules or polymer-based delivery systems, aligning with increasing regulatory and consumer demand for environmentally responsible ingredients.

### Limitations

Despite these strengths, several limitations should be acknowledged. This was an exploratory pilot study with a small sample size, and while statistically significant differences were observed, the study was not powered to detect subtle effects or to perform subgroup analyses based on age, skin type, or olfactory sensitivity. The study population consisted solely of female participants, which may limit generalizability, as sex-related differences in olfactory perception have been reported. In addition, fragrance intensity was assessed over an 8 h period under controlled conditions; longer-term studies assessing day-long wear, repeated application, and real-world environmental exposure (e.g., heat, humidity, and physical activity) would provide further insight into performance. There was a baseline imbalance; however, the within-subject design mitigates its impact.

Another consideration is that fragrance perception does not equate directly to fragrance composition or release kinetics. While the sustained sensory effect observed strongly suggests controlled release from SpECs, future studies incorporating analytical techniques such as headspace gas chromatography–mass spectrometry could quantitatively characterize volatile release profiles and correlate them with sensory outcomes. Furthermore, while in vitro cytotoxicity testing supports safety, formal dermatological testing, including skin irritation, sensitization, and long-term tolerability studies, will be essential prior to large-scale clinical or commercial application. Further studies with lesser loading than 100% should be undertaken to determine if the loading is directly related to the duration of action of the fragrance.

## 6. Conclusions

In conclusion, this study provides the first human evidence that sporopollenin exine capsules can effectively prolong fragrance perception when incorporated into topical fragrance formulations, without evidence of cytotoxicity. These findings extend the application of SpEC technology beyond pharmaceutical and nutraceutical delivery into the field of fragrances and cosmetics. With further validation in larger, diverse populations and expanded safety and mechanistic studies, SpECs have the potential to represent a novel, natural, and sustainable platform for sustained-release fragrance delivery.

## Figures and Tables

**Table 1:** Summary statistics of fragrance intensity scores (participant-rated).

Summary Statistics—Fragrance Intensity								
Group	Timepoint	N Obs	Mean	Std Dev	Median	Minimum	Maximum	Quartile Range
SpECs	0 h	10	8.2	2.2	9.0	3.0	10.0	2.0
	2 h	10	7.0	1.3	7.0	4.0	9.0	1.0
	4 h	10	5.1	1.1	5.0	3.0	7.0	1.0
	8 h	10	3.7	1.3	4.0	2.0	6.0	1.0
Control	0 h	10	7.2	1.4	8.0	5.0	9.0	2.0
	2 h	10	5.9	0.99	6.0	4.0	7.0	2.0
	4 h	10	4.1	1.1	4.0	2.0	6.0	1.0
	8 h	10	2.0	0.9	2.0	1.0	4.0	1.0

**Table 2:** Summary statistics of fragrance intensity scores (reviewer rated).

Summary Statistics—Fragrance Intensity								
Group	Timepoint	N Obs	Mean	Std Dev	Median	Minimum	Maximum	Quartile Range
SpECs	0 h	10	8.6	1.3	9.0	6.0	10.0	1.0
	2 h	10	7.4	0.5	7.0	7.0	8.0	1.0
	4 h	10	5.9	0.6	6.0	5.0	7.0	0
	8 h	10	4.3	0.9	4.0	3.0	6.0	1.0
Control	0 h	10	8.1	0.7	8.0	7.0	9.0	1.0
	2 h	10	6.3	0.9	6.0	5.0	8.0	1.0
	4 h	10	4.7	0.9	5.0	3.0	6.0	1.0
	8 h	10	2.5	0.9	2.0	1.0	4.0	1.0

## Data Availability

The original contributions presented in this study are included in the article/Supplementary Material. Further inquiries can be directed to the corresponding author.

## Supplementary Materials

The following supporting information can be downloaded at: https://www.mdpi.com/article/10.3390/pharmaceutics18050609/s1, Figure S1: Scanning electron micrograph of SpECs, showing the highly uniform spherical structure of *Lycopodium clavatum* with characteristic serrated coronate features (Micrograph acquired at 2000x magnification); Figure S2: FTIR spectrum of untreated (raw) *Lycopodium clavatum* spores. A broad absorption band at 3400–3300 cm^−1^ is attributed to O-H stretching vibrations of hydroxyl groups. C-H bands are observed at 2925 and 2854 cm^−1^. Thes indicate the presence of long-chain aliphatic structures. The prominent absorption at 1735 cm^−1^ is assigned to ester carbonyl (C=O) stretching vibrations. The bands at 1650 and 1540 cm^−1^ are due to amide I and amide II vibrations; Figure S3: FTIR spectrum of SpEC. The treated spores show recued intensity of the carbonyl stretching at 1700 cm^−1^ indicating removal of ester-containing compounds, fatty acids, proteins or pigments. The OH stretching band at 3400 cm^−1^ is more intense indicating increased exposure of hydroxyl groups due to hydrolysis and removal of surface biomolecules. Despite these significant changes other characteristic sporopollenin-associated bands remain preserved indicting that the chemically resistant exine framework is retained after the chemical treatment.

## References

[1] Diego-Taboada A., Beckett S.T., Atkin S.L., Mackenzie G. (2014). Hollow pollen shells to enhance drug delivery. Pharmaceutics.

[2] Niinuma S.A., Khudair A.D., Habib H., Khudair A.D., MacKenzie G., Atkin S.L., Butler A.E. (2024). Unearthing nature’s remedy: An exploration into Lycopodium’s medicinal and therapeutic potential. Appl. Mater. Today.

[3] Zhao Y., Wang Y., Zhang Z., Li H. (2023). Advances in controllable release essential oil microcapsules and their promising applications. Molecules.

[4] Mikhael A., Jurcic K., Schneider C., Karr D., Fisher G.L., Fridgen T.D., Diego-Taboada A., Georghiou P.E., Mackenzie G., Banoub J. (2020). Demystifying and unravelling the molecular structure of the biopolymer sporopollenin. Rapid Commun. Mass. Spectrom..

[5] Thomasson M.J., Baldwin D.J., Diego-Taboada A., Atkin S.L., Mackenzie G., Wadhawan J.D. (2010). Electrochemistry and charge transport in sporopollenin particle arrays. Electrochem. Commun..

[6] Potroz M.G., Mundargi R.C., Gillissen J.J., Tan E.L., Meker S., Park J.H., Jung H., Park S., Cho D., Bang S.I. (2017). Plant-based hollow microcapsules for oral delivery applications: Toward optimized loading and controlled release. Adv. Funct. Mater..

[7] Mackenzie G., Boa A.N., Diego-Taboada A., Atkin S.L., Sathyapalan T. (2015). Sporopollenin, the least known yet toughest natural biopolymer. Front. Mater..

[8] Barrier S., Diego-Taboada A., Thomasson M.J., Madden L., Pointon J.C., Wadhawan J.D., Beckett S.T., Atkin S.L., Mackenzie G. (2011). Viability of plant spore exine capsules for microencapsulation. J. Mater. Chem..

[9] Wakil A., Mackenzie G., Diego-Taboada A., Bell J.G., Atkin S.L. (2010). Enhanced bioavailability of eicosapentaenoic acid from fish oil after encapsulation within plant spore exines as microcapsules. Lipids.

[10] Diego-Taboada A., Sathyapalan T., Courts F., Lorch M., Almutairi F., Burke B.P., Harris K., Kruusmägi M., Walther T., Booth J. (2022). Spore exines increase vitamin D clinical bioavailability by mucoadhesion and bile triggered release. J. Control. Release.

[11] Deng J., Zhao Z., Yeo X.Y., Yang C., Yang J., Ferhan A.R., Jin B., Oh C., Jung S., Suresh S. (2024). Plant-Based Shape Memory Cryogel for Hemorrhage Control. Adv. Mater..

[12] Aylanc V., Ertosun S., Estravís M., Dávila I., Sánchez Reyes E., Vale N., Freire C., Vilas-Boas M. (2026). Investigating human IgE antibody interactions with pollen-derived sporopollenin biopolymers: Immunoreactivity profiling for the rational design of structurally robust and biocompatible biomaterials. Biomed. Mater..

[13] Harris T., Wenthur C., Diego-Taboada A., Mackenzie G., Corbitt T., Janda K. (2016). Lycopodium clavatum exine microcapsules enable safe oral delivery of 3, 4-diaminopyridine for treatment of botulinum neurotoxin A intoxication. Chem. Commun..

[14] Atkin S., Mackenzie G., Beckett S. (2005). Patent ‘Dosage Form’.

[15] Birkett M.A., Day S.J. (1994). Internal pilot studies for estimating sample size. Stat. Med..

[16] Behrendt H., Becker W.-M. (2001). Localization, release and bioavailability of pollen allergens: The influence of environmental factors. Curr. Opin. Immunol..

[17] Diego-Taboada A., Maillet L., Banoub J.H., Lorch M., Rigby A.S., Boa A.N., Atkin S.L., Mackenzie G. (2013). Protein free microcapsules obtained from plant spores as a model for drug delivery: Ibuprofen encapsulation, release and taste masking. J. Mater. Chem. B.

[18] Hasan A., Stanley J., Moin A.S.M., Begam H., Waris S., Abdulhadi M., Greish K., Boa A.N., Kumar M.P., Deen G.R. (2026). In vitro and in vivo assessment of sporopollenin exine capsule preparations (SpECs) from Lycopodium clavatum spores. RSC Adv..

[19] Binks B.P., Boa A.N., Kibble M.A., Mackenzie G., Rocher A. (2011). Sporopollenin capsules at fluid interfaces: Particle-stabilised emulsions and liquid marbles. Soft Matter.

[20] Diego-Taboada A., Cousson P., Raynaud E., Huang Y., Lorch M., Binks B.P., Queneau Y., Boa A.N., Atkin S.L., Beckett S.T. (2012). Sequestration of edible oil from emulsions using new single and double layered microcapsules from plant spores. J. Mater. Chem..

