# Supplementary material for: Double-Blind Placebo-Controlled Randomized Study of Sporopollenin Exine (SpEC) Fragrance Encapsulation
Source: Pharmaceutics. 2026 May 17;18(5):609. doi: 10.3390/pharmaceutics18050609 (PMC13210823; doi:10.3390/pharmaceutics18050609)
Supplement: Supplementary file 1 [file pharmaceutics-18-00609-s001.zip › pharmaceutics-4294329-supplementary.pdf]

Supplementary information

Double Blind Placebo Controlled Randomised Study of Sporopollenin Exine (SpECS) Fragrance Encapsulation

Mariam Murad [1], Pearl Wasif [1], Laura Dempsey [2], G. Roshan Deen [3], Alexandra E Butler [1]* and Stephen L. Atkin [1]

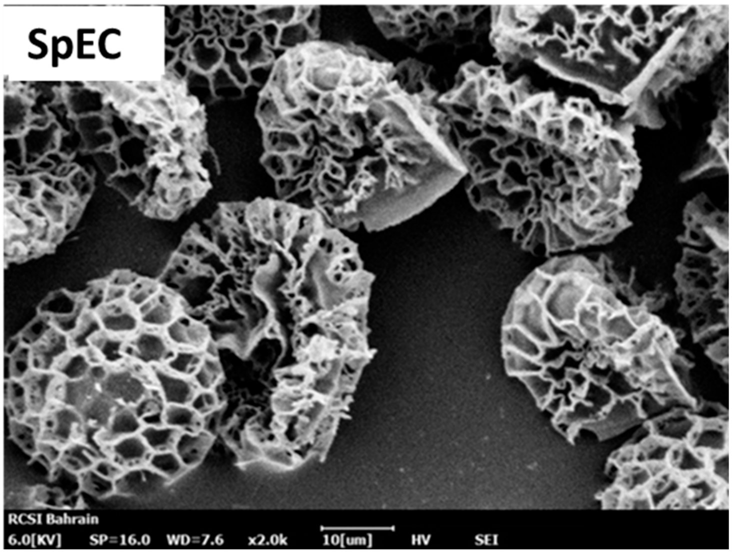

Figure S1. Scanning electron micrograph of SpECs, showing the highly uniform spherical structure of Lycopodium clavatum with characteristic serrated coronate features (Micrograph acquired at 2000x magnification).

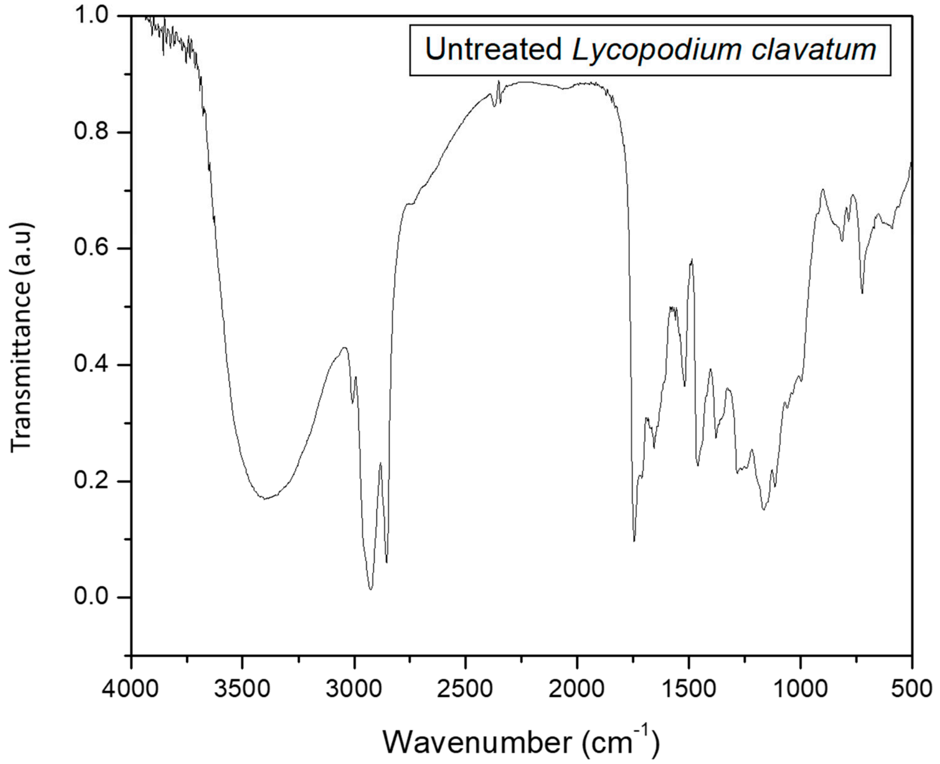

Figure S2. FTIR spectrum of untreated (raw) *Lycopodium clavatum* spores. A broad absorption band at 3400-3300 cm^{-1} is attributed to O-H stretching vibrations of hydroxyl groups. C-H bands are observed at 2925 and 2854 cm^{-1}. Thes indicate the presence of long-chain aliphatic structures. The prominent absorption at 1735 cm^{-1} is assigned to ester carbonyl (C=O) stretching vibrations. The bands at 1650 and 1540 cm^{-1} are due to amide I and amide II vibrations.

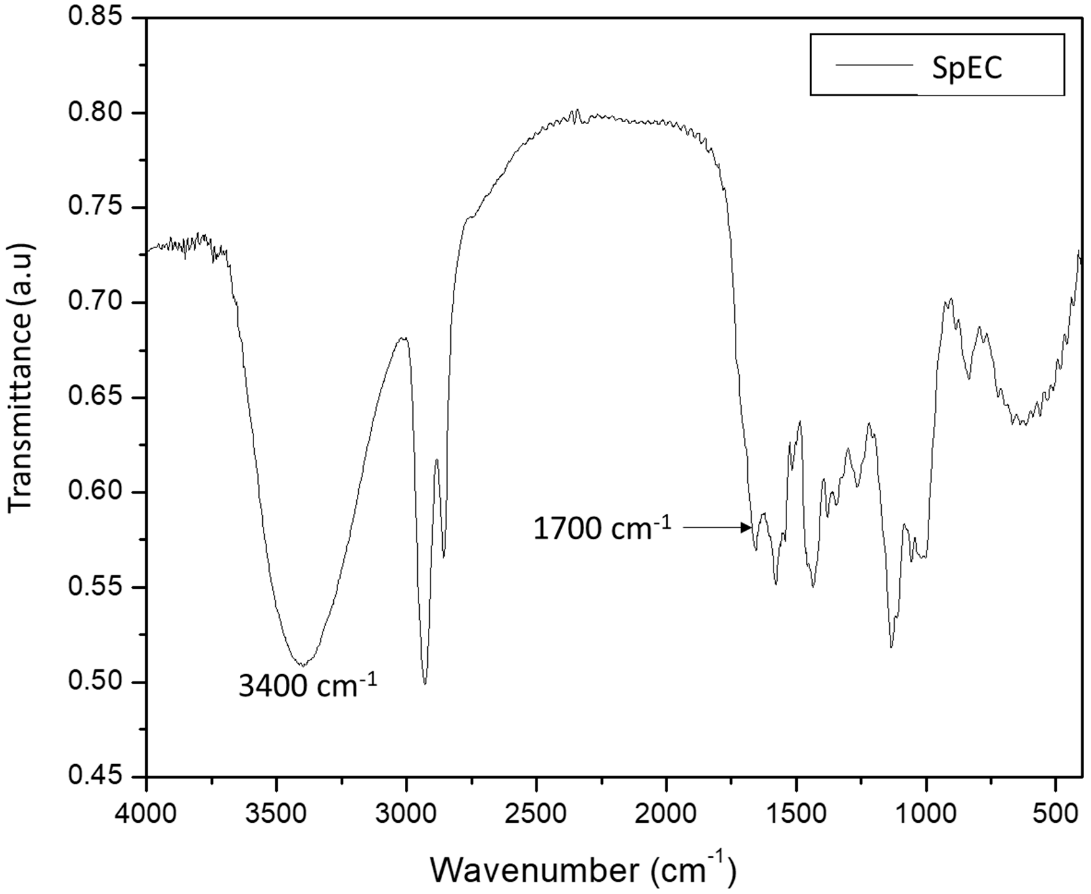

Figure S3. FTIR spectrum of SpEC. The treated spores show recued intensity of the carbonyl stretching at 1700 cm^{-1} indicating removal of ester-containing compounds, fatty acids, proteins or pigments. The OH stretching band at 3400 cm^{-1} is more intense indicating increased exposure of hydroxyl groups due to hydrolysis and removal of surface biomolecules. Despite these significant changes other characteristic sporopollenin-associated bands remain preserved indicting that the chemically resistant exine framework is retained after the chemical treatment.
